# Removal of Parasite Transmission Stages from Berries Using Washing Procedures Suitable for Consumers

**DOI:** 10.3390/foods10020481

**Published:** 2021-02-23

**Authors:** Tamirat T. Temesgen, Lucy J. Robertson, Vetle M. Stigum, Kristoffer R. Tysnes

**Affiliations:** Laboratory of Parasitology, Department of Paraclinical Sciences, Faculty of Veterinary Medicine, Adamstuen Campus, Norwegian University of Life Sciences, P.O. Box 369 Sentrum, 0102 Oslo, Norway; lucy.robertson@nmbu.no (L.J.R.); vetle.malmer.stigum@nmbu.no (V.M.S.); Kristoffer.tysnes@nmbu.no (K.R.T.)

**Keywords:** berries, washing, removal, contamination, foodborne parasite, *Cyclospora*, *Giardia*, *Cryptosporidium*

## Abstract

Due to the delicate nature of berries and the reduced shelf-life once washed, producers usually do not wash berries. Therefore, consumers are expected to wash the berries prior to consumption, and this might be a more effective way of infection prevention. However, the efficacy of consumer berry-washing procedures in removing the parasite contaminants from the berries surface has not been investigated. The aim of the present study was, therefore, to compare the efficacy of three different washing techniques in removing parasite contaminants. Three alternatives to washing berries before consumption were compared on berries artificially contaminated with oo/cysts of *Cyclospora cayetanensis*, *Cryptosporidium parvum*, and *Giardia duodenalis*. The results show that simple washing of berries under the cold tap for 1 min could remove on average at least 80% of the parasites, except for *C. cayetanensis*, which seems to be stickier than both *G. duodenalis* and *C. parvum*. The percent removal was slightly lower for raspberries as compared to blueberries. Although the differences are expected, a relevant result of the study is that washing contaminated berries prior to consumption by the consumer removes a considerable proportion of parasites and thereby lowers the risk of ingesting parasites’ transmission stages.

## 1. Introduction

Foodborne illnesses are frequently caused by consumption of food contaminated with pathogens and often have symptoms of diarrhoea and/or vomiting. Among the pathogens are various bacteria, viruses, and parasites; the latter of these have received less attention regarding prevention and control [1]. This is not because parasites are less pathogenic, but probably reflects various factors, such as lack of awareness among many physicians, an absence of appropriate laboratory methods for detection, and challenges related to source attribution due to the long incubation period of many parasitic diseases [2].

Foodborne parasites (FBP) remain a public health problem worldwide, affecting the health and wellbeing of people. Although transmission of FBP could be prevented, nevertheless, millions of people are infected every year with resulting diseases ranges from mild to severe, and even deaths [3]. Estimates show that more than 90 million people were ill due to FBP in 2010, resulting in more than 50 thousand deaths, and loss of more than 7 million healthy life years [3].

A wide range of parasites can potentially be transmitted via contaminated fresh produce, including berries, and some have been implicated in outbreaks of disease. Three protozoan parasites, *Cyclospora cayetanensis*, *Cryptosporidium parvum*, and *Giardia duodenalis*, were considered in the present study. These parasites, which may cause gastrointestinal disease when ingested, are known for their robust transmission stages that are able to withstand harsh environmental conditions and have prolonged survival in the environment. All three parasites follow a direct life cycle that is completed in a single host. *C. cayetanensis* is assumed to infect only human hosts [4] although it remains uncertain as to whether non-human primates, dogs, cattle, and chickens could serve as hosts of the parasite [5]. In contrast, *C. parvum* is zoonotic, with some outbreaks associated with contamination of fresh produce with oocysts from calves [6], and some assemblages of *G. duodenalis* have zoonotic potential.

There have been repeated outbreaks of foodborne cyclosporiasis in the U.S., mostly associated with consumption of contaminated fresh produce [4,5]. For other FBP, e.g., *Cryptosporidium* and *Giardia*, the role of fresh produce as vehicle of transmission is less clear. However, there are several reports of foodborne cryptosporidiosis outbreaks. The identified vehicles of infection in these outbreaks include fresh produce, apple cider/juice and dairy products [7].

Many parasite transmission stages are sticky in nature and this might facilitate attachment to different transmission vehicles, thereby enhancing the chance to infect new hosts. According to one study on *Cryptosporidium* oocysts spiked onto apples, it was not possible to attain complete removal of the oocysts using the elution methods commonly used for detection purposes [8]. Using scanning electron microscopy, Macarisin et al. (2010) revealed a filamentous matrix between the parasite and the apple surface, but it was not clear if this was derived from the oocyst or from the apple. The oocysts of *C. cayetanensis* are believed to be even more sticky than *Cryptosporidium* oocysts, probably due to their specific adhesins [9].

In addition to the sticky nature of the parasites’ transmission stages, the surface morphology of some berries enables the adherence of parasites. Raspberries have been shown to retain more *T. gondii* oocysts than blueberries [10]. In that study, mice were fed different types of berries inoculated with as few as 10 oocysts and only mice that were fed contaminated raspberries developed patent infections. This was speculated to be due to the hairy projections on the surface of raspberries that contributed to greater retainment of the parasites than the smoother surfaces of blueberries [10].

Several studies have been conducted to assess the parasite contamination of berries and have indicated that berries have the potential to act as vehicles for transmission of FBP. For example, a study conducted in Bogota, Colombia indicated that 5% (6 out of 120) strawberry samples were contaminated with *Toxoplasma gondii* DNA and 1 sample was contaminated with *Cyclospora* cayetanensis DNA [11]. Earlier studies using a microscopy-based ISO procedure for detection have also shown berry contamination with the transmission stages of *Cryptosporidium* and *Giardia* [12,13]. Although the infectivity of the parasites detected on berries has not been determined, given the robustness and the low infectious dose of the parasite stages makes such findings of public health relevance.

Due to the delicate nature of berries and their reduced shelf-life once washed, berries are usually not washed prior to sale. Consumers are therefore advised to wash berries before consumption. Methods of washing berries are likely to differ among consumers, with some not washing berries at all, others rinsing briefly under the tap, and others using more rigorous washing [14]. However, the efficacy of different berry washing approaches at removing parasite contaminants from berry surfaces has, to our knowledge, not been investigated. The aim of the present study was therefore to compare removal of different parasite transmission stages from berries using three different consumer-recommended washing techniques.

## 2. Materials and Methods

### 2.1. Sample Preparation

#### 2.1.1. Parasites

In the present study, *G. duodenalis* cysts, *C. cayetanensis* oocysts, and *C. parvum* oocysts were used for the comparative evaluation of the removal efficacy of three different washing techniques, as described in Section 2.2. The *G. duodenalis* cysts were purchased from Waterborne, Inc. (New Orleans, LA, USA); *C. parvum* oocysts were purchased from Bunch Grass Farm (Deary, ID, USA); *C. cayetanensis* oocysts were kindly provided by Dr. Kristin Elwin (Public Health Wales, UK). The concentrations of each species were estimated using KOVA^®^ Glasstic^®^ Slide 10 Microscope Slide (VWR, Oslo, Norway). The oo/cysts of *Cryptosporidium* and *Giardia* were used within 6 months. However, the oocysts of *C. cayetanensis* were older than 12 months at the time of the experiment.

#### 2.1.2. Berry Samples and the Spiking Protocol

Fresh blueberries and raspberries used in this study were kindly provided by BAMA and Coop Norge. About 100 g of blueberries and raspberries were weighed into plastic boxes and spiked with all the three parasite stages. A separate suspension of 50 µL that contained approximately 3 × 10^5^ cysts of *G. duodenalis*, 5 × 10^5^ oocysts of *C. cayetanensis*, and 2 × 10^6^ oocysts of *C. parvum* were used for contaminating the berries. The spiking with each suspension was done on five different spots (each spot of 10 µL) on the surface of berries. A high number of oo/cysts were used for these spiking studies in order to increase the likelihood that oo/cysts unremoved by washing would be detectable and quantifiable. The spiked berries were kept at room temperature for 3 h and then overnight in the refrigerator with the lids closed. The berries were then washed by either of the three washing alternatives described below (Section 2.2). About 100 g of unspiked berries were also processed according to Section 2.4.

### 2.2. Three Alternatives to Washing of Berries

The procedures for washing the berries were adapted from online sites aimed at berry consumers (https://www.wikihow.com/Clean-Blueberries (accessed on 15 January 2021); https://www.wikihow.com/Clean-Raspberries (accessed on 15 January 2021)). The three methods are described below.

#### 2.2.1. Washing under Running Water (RW)

The berries were placed in a sieve (strainer) and rinsed under a gentle stream of cold tap water for 1 min, with the sieve moved gently so that all the berries were rinsed. After rinsing, the sieve was shaken gently to remove excess water droplets.

#### 2.2.2. Washing using Vinegar (VG)

One part vinegar was mixed with three parts cold tap water (1.75% acetic acid final concentration) in a bowl of sufficient size to hold the berries. The berries were placed in the bowl and stirred for 1 min by hand, swishing the berries around in the bowl. The bowl’s contents were then poured into a sieve and rinsed under a gentle stream of cold water for 30 s. After rinsing, the sieve was shaken gently to remove excess water droplets.

#### 2.2.3. Washing Followed by Salad Spinner (SP)

The salad spinner’s bowl was filled with cold water with the spinner “cage” inside it. The berries were placed in the salad spinner cage and stirred for 1 min by hand, swishing the berries around the bowl by hand. The cage was lifted out of the bowl, which was then emptied of washing liquid, before the cage being replaced in the bowl and the berries spun for 10 s twice in alternate directions to remove excess water droplets.

### 2.3. Experimental Design

**Figure 1:**
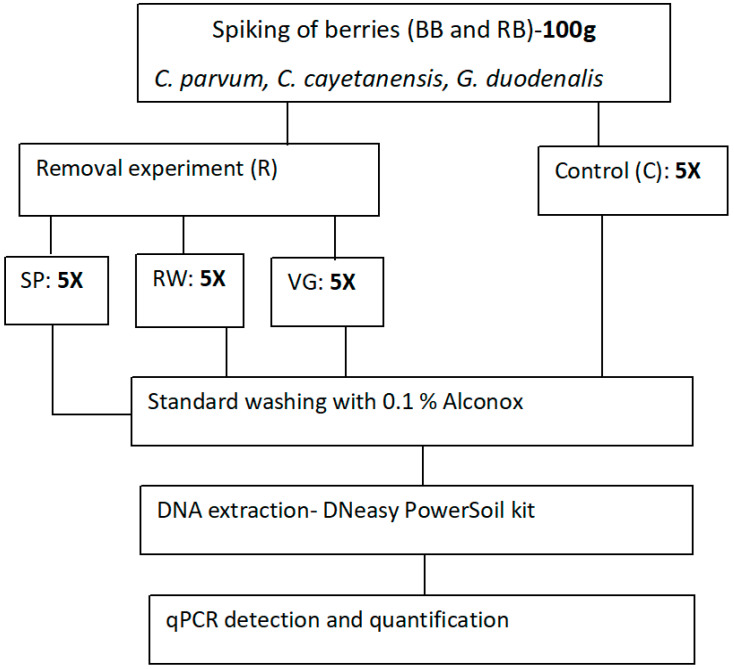
The experimental design for comparative evaluation of the removal efficiency of 3 methods for washing berries prior to consumption. Key: BB—blueberry, RB—raspberry, RW—running water, VG—vinegar, SP—salad spinner.

Five independent replicates of spiked berries were analysed per washing technique, including the control group, which was not subject to any of the three washing alternatives but directly processed for DNA extraction and qPCR analyses. The experimental design for this study is presented in Figure 1. In addition, unspiked berries were also included as a control to ensure that any amplifications are from those parasites that had been experimentally spiked.

### 2.4. Sample Processing for DNA Extraction

After the berry samples were washed with either of the three washing alternatives, the samples were processed for DNA extraction as previously described [15,16]. Briefly, the washed berries were transferred into plastic boxes to which 200 mL of 0.1% Alconox^TM^ (Alconox Inc., White Plains, NY, USA) was added. The boxes were then placed on an automatic shaker (Heidolph Vibramax 100); raspberry samples were shaken at 300 rpm for 10 min, whereas blueberry samples were shaken at 600 rpm for 10 min.

The rinsate from the boxes was then transferred into four 50 mL tubes for concentration by centrifugation at 1690× *g* for 10 min and the supernatant removed by vacuum suction, leaving 10 mL of the sediment. The pooled sediment was centrifuged at 3803× *g* for 10 min with a deceleration break set to 6 (on a scale of 0–9). The remaining supernatant and pellet (about 1.5 mL) was transferred 2.0 mL tubes and further concentrated to 250 µL by centrifugation at 13,000× *g* for 5 min. The final sediment was immediately subjected to DNA extraction as described below (Section 2.5).

### 2.5. DNA Extraction

DNA extraction was performed with the DNeasy PowerSoil kit (Qiagen, Oslo, Norway) protocol, with slight modifications. Briefly, 60 µL of the lysis solution (solution C1) and 250 µL of the concentrated sediments from the berry washes were mixed in the PowerBead tube and then subjected to bead-beating to break the oo/cyst walls, thereby facilitating the release of DNA. Bead-beating was performed using FastPrep-24 5G™ High Speed Homogeniser (MP Biomedicals, Illkirch Cedex, France) in two cycles of 4 m/s for 60 s with 45 s pause between the cycles. The lysate was then centrifuged at 10,000× *g* for 1 min at room temperature, and 500 µL of the supernatant used for the subsequent step in the protocol. The DNA was eluted in 50 µL of the elution solution (solution C6) and stored at −20 °C. Bead beating has been previously found effective for *Giardia* cysts [17] and *Cryptosporidium* oocysts [18].

### 2.6. Detection and Quantification of Parasites Using Real-Time PCR (qPCR)

The PCR was performed in a 0.3 mL PCR plate without skirt (Sarstedt, Oslo, Norway) and the platform used for the qPCR was Agilent’s Stratagene Mx3005P. ROX was used as a reference dye against which the target fluorescence data were normalized. Every qPCR run was performed in triplicate with no-template and positive controls (gDNA isolated from the same number of parasites as used in the spiking experiments) included.

For the comparative evaluation of different washing techniques in removing *Cryptosporidium* and *Giardia* from contaminated berries, qPCR protocols that had been previously described for the detection and quantification of *Cryptosporidium* spp. [19] and *G. duodenalis* [20] were used with slight modifications.

The qPCR protocol for *Cryptosporidium* was performed in a total volume of 25 µL that included 5 µL of template DNA, 0.6 µM of each primer and 80 nM of the Probe, and 12.5 µL of 2× KiCqStart^®^ Probe qPCR ReadyMix™, low ROX^TM^ (Sigma-Aldrich, Oslo, Norway). The oligos included forward primer JF1/2 which was prepared in 1:1 mixture of JF1 (5′-AAGCTCGTAGTTggatTTCTG-3′) and JF2 (5′-AAGCTCGTAGTTaatcTTCTG-3′), the reverse primer JR (5′-TAAGGTGCTGAAGGAGTAAGG-3′), and the probe JT2 (5′- TCAGATACCGTCGTAGTCT-3′).

For *Giardia*, 0.4 µM of each primer *Giardia*-80F (5′-GACGGCTCAGGACAACGGTT-3′) and *Giardia*-127R (5′-TTGCCAGCGGTGTCCG-3′), and 0.16 µM of the probe *Giardia*-105T (FAM-5′-CCCGCGGCGGTCCCTGCTAG-3′-MGBEQ), 12.5 µL of 2× KiCqStart^®^ Probe qPCR ReadyMix™, low ROX^TM^ (Sigma-Aldrich, Oslo, Norway), and 5 µL of the DNA template was combined in a total volume of 25 µL. The thermal cycling included an initial denaturation at 95 °C for 3 min followed by 45 cycles of denaturation at 95 °C for 15 s and annealing at 60 °C for 60 s and extension at 72 °C for 30 s.

The detection and quantification of *C. cayetanensis* was performed according to a previously published protocol [15]. Briefly, the qPCR contained 0.5 µM of each primer CyITS1_TT-F ATGTTTTAGCATGTGGTGTGGC and CyITS1_TT-R GCAGCAACAACAACTCCTCATC, 0.15 µM of the probe CyITS1_TT-P HEX-TACATACCCGTCCCAACCCTCGA-MGBEQ, 10 µL of 2× KiCqStart^®^ Probe qPCR ReadyMix™, low ROX^TM^ (Sigma-Aldrich, Oslo, Norway), and 2 µL of template in 20 µL reaction volume. The thermal cycling included an initial denaturation at 95 °C for 3 min followed by 45 cycles of denaturation at 95 °C for 15 s and combined annealing and extension at 60 °C for 30 s.

Standard curves were prepared from five-point 10-fold serial dilutions of the DNA extracted from the same number of parasites used for spiking the berries, i.e., 3 × 10^5^ cysts of *G. duodenalis*, 5 × 10^5^ oocysts of *C. cayetanensis*, and 2 × 10^6^ oocysts of *C. parvum*. The standard curve was included in each run to estimate the relative quantity of DNA recovered from each sample.

### 2.7. Statistical Analysis

The fluorescence data were collected automatically by Stratagene Mx3005P. The raw fluorescence intensity was evaluated against the recommended range of the instrument by using the multicomponent view. The mean C_q_ values of samples run in triplicates was used for calculations. The mean C_q_ values were automatically converted to estimated number of oo/cysts in the sample, by MxPro^TM^ qPCR software, using the standard curve prepared from known amounts of each parasite. The estimated number of oo/cysts with their respective washing technique category were then exported to Excel sheet (Microsoft^®^ Office Excel^®^ 2010) for further statistical analysis. The removal efficiency of different washing methods was estimated according to equation 1 using Microsoft Office Excel, where extrapolation from the standard curves are used as estimates of the parasite numbers after washing; the numbers used in the standard curve having been determined initially by microscopy counts.

(1) $$\%\;removal\;=\left[1-\left(\frac{estimated\;no.\;of\;parasite\;detected\;after\;washing}{estimated\;no.\;of\;parasite\;spiked}\right)\right]\times 100$$

JMP^®^ Pro version 15 software (SAS institute Inc.) was used for calculating the 95% CI of the median percentage removal of each washing method. Quantile regression was performed to evaluate the significance of the differences between each washing methods and parasites.

## 3. Results

The qPCR methods used for the detection and quantification of *C. cayetanensis*, *C. parvum*, *and G. duodenalis* showed acceptable performance, with efficiencies ranged between 91.5% and 108.4% (Supplementary File 1). The linearity of the qPCR methods was also shown to be acceptable, as shown by the coefficient of determination (r^2^ > 0.99) (Supplementary File 1).

The comparison of three washing techniques that included washing under running water (RW), using salad spinner (SP), and vinegar (VG) showed significant differences in terms of their removal efficiency of parasites from contaminated berries. The overall removal efficacy, in increasing order, was RW < SP < VG (see Supplementary File 2 for more detail). Compared to the two other washing methods, washing berries under tap water (RW) was significantly less efficient at removing parasites (Wald χ² = 7.9, *p* = 0.005).

**Figure 2:**
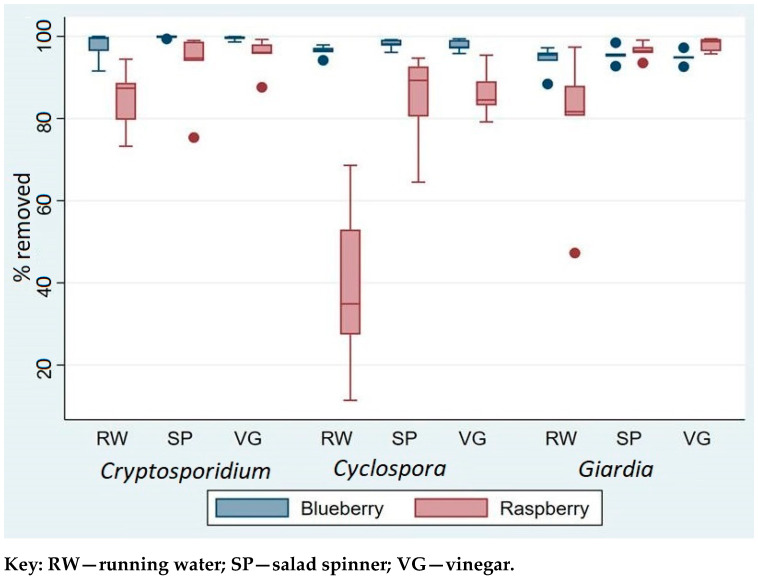
Box plot of the percentage removal of parasites by three washing techniques. Dots represent outliers.

Nevertheless, for *Cryptosporidium* and *Giardia*, simple washing of berries under the cold tap for 1 min could remove on average at least 80% of the parasites. For *C. cayetanensis*, which seems to be stickier than both *G. duodenalis* and *C. parvum*, removal by rinsing under tap water (RW) was lower, ranging from 11.4–68.6% (Figure 2).

The findings also showed that contaminant parasites were easier to remove from blueberries than raspberries. The overall removal of parasites was significantly lower on raspberries than on blueberries (Wald χ^2^ = 15.4, *p* < 0.0001), regardless of method. Whereas between 94 and 97% of the different parasites were removed from blueberries by washing under running water, for raspberries the removal was lower, ranging from about 84% for *Cryptosporidium* oocysts to 39% for *Cyclospora* oocysts (Table 1).

Although RW was least effective at removing contamination for all three parasites, this was especially so for *Cyclospora*. Although the combined data showed no statistically significant difference among the three parasites (Wald χ^2^ = 1.4, *p* = 0.232), separate analysis of the removal efficacy from raspberries showed that the percentage removal of *Cyclospora* was significantly lower than for *Cryptosporidium* and *Giardia* (Wald χ^2^ = 21.2, *p* < 0.0001).

## 4. Discussion

To the best of our knowledge, there have been no previous studies conducted to assess the efficacy of consumer-friendly washing methods in removing FBP from berry surfaces. However, some studies have considered the evaluation of various consumer-friendly washing methods in reducing bacteria on fresh produce [21,22]. One study showed that total aerobic count and Enterobacteriaceae count was reduced by over 65% by a single rinse, this did not affect *Escherichia coli* O157:H7 [22]. However, contrasting results were provided in another study that demonstrated 1–3 log reductions were shown for various bacteria (*E. coli* O157:H7, *Listeria monocytogenes*, and *Salmonella enterica*) [21]. Furthermore, washing of fruits and vegetables prior to displaying it for sale in Arba Minch, Ethiopia was shown to decrease contamination with parasites significantly [23]. In contrast, a study on removal of *C. parvum* oocysts from apple surfaces showed that they attached firmly and could only be removed by rigorous manual washing [8].

The present study compared the efficacy of three different washing techniques in removing *C. cayetanensis* and *C. parvum* oocysts, and *G. duodenalis* cysts from artificially contaminated raspberries and blueberries. The overall findings of the study were that on average at least 80% of the parasites (except *C. cayetanensis*) were removed from either type of berry by any of the three washing techniques used.

However, *Cyclospora* oocysts were notably more difficult to remove from the surfaces of raspberries. This probably reflects both the nature of raspberries’ surface and the biological makeup of the oocysts. The stickiness of *Cyclospora* oocysts has previously been found to be stronger than that of *Cryptosporidium* oocysts and *Giardia* cysts, probably due to their specific adhesins, the origin of which have not been fully explored [24]. In addition to the sticky nature of the oocysts, the surfaces of raspberries probably enable firmer adherence of the parasites. The findings of significant differences in removal between blueberries and raspberries in this study were expected and corroborate reports from previous studies. It has previously been shown that raspberries retained more oocysts of *T. gondii* than blueberries in a study in which mice fed with raspberries inoculated with just 10 oocysts became infected, but this did not occur for blueberries that were similarly spiked [10]. This was speculated to be due to the hairy projections (pistils) on the surface of raspberries that contribute to greater retainment of the parasites than the smoother surfaces of blueberries [10]. It is expected that although thorough washing may remove oo/cysts, it is difficult to clean all berries adequately [25].

According to the findings of the present study, there was no significant difference between the samples washed by vinegar solution and those washed using water but drained by salad spinner. However, there was significant difference between washing under running tap water and vinegar solution. It is not clear whether the difference was due to the vinegar solution per se or due to the manual agitation, with the berries swished around in the bowl by hand. Organic acids (including acetic, lactic, malic, tartaric, and citric acids) have been investigated as natural sanitizers for fresh produce [26], and have been shown to inactivate cells by the pH decrease damaging membranes and key enzyme functions [27]. It is possible that with fewer bacteria on berry surfaces providing a matrix for parasite adhesion, removal by washing is more successful. Indeed, undiluted vinegar solution has also been reported to inactivate *Giardia* cysts [28], although temperature, contact time, and concentration appear to be important considerations [27].

In order to maintain the freshness of berries, producers use cold chain during storage and shipment, which also includes using modified atmosphere to reduce deterioration, particularly for international transport [29]. A potential negative side effect of these conditions is that they also favour survival of foodborne parasites [20]. It has previously been reported that the survival of *Giardia* cysts, which are less robust than *Cryptosporidium* oocysts, on lettuce leaves was improved when refrigerated [30].

As fresh berries are often consumed without further processing, it is important for consumers to be encouraged to implement measures to reduce the likelihood of ingesting parasite transmission stages. Our results indicate that even simple washing of berries contaminated with parasite transmission stages removes a considerable proportion of the parasite load. The washing procedures that we used for this study are described on an easily accessible consumer site and are simple, inexpensive, and quick to perform. Links to such sites could be provided by the berry industry, and other stakeholders, to remind consumers of the benefits of washing berries before eating them.

The findings of the present study might be affected by the fact that the oo/cysts used for spiking were older than 3 months. It is known that the age of the parasites stages affects their adhesion characteristics, i.e., the older the parasites the stronger their attachment to environmental matrices [31]. It might be interesting to conduct experiments to study the effect of differences in the age of oo/cysts on the removal efficacy of the washing alternatives by using fresh and old oo/cysts.

In conclusion, the findings of the present study showed that the choice of washing technique could significantly affect the removal of certain parasites. Although simple rinsing under running water removes a substantial amount of contaminating parasite transmissions stages, for some parasites, such as *C. cayetanensis*, on some types of berries, such as raspberries, removal may be enhanced by a more vigorous washing step, prior to consumption.

## Figures and Tables

**Table 1:** Percentage removal of parasites from raspberries and blueberries after washing with three different washing techniques in five replicates.

**Percent Removal of Parasites from Raspberries**								
Washing alternative	*C. parvum*					Median	95% CI	
Running water	94.45	87.37	88.69	73.26	79.69	87.37	73.25	94.44
Vinegar	87.61	99.24	95.71	98.06	96.09	96.09	87.61	99.24
Salad spinner	94.01	94.7	98.71	98.99	75.36	94.70	75.36	98.99
	*G. duodenalis*							
Running water	87.97	80.67	97.37	47.26	81.67	81.67	47.26	97.37
Vinegar	98.71	99.39	96.39	99.36	95.73	98.71	95.73	99.39
Salad spinner	96.43	95.92	97.44	99.08	93.52	96.43	93.51	99.08
	*C. cayetanensis*							
Running water	68.61	52.97	27.41	11.41	34.88	34.88	11.41	68.61
Vinegar	84.52	95.41	79.18	89.07	83.19	84.52	79.17	95.41
Salad spinner	92.67	89.28	80.48	94.69	64.51	89.28	64.51	94.68
**Percent removal of parasites from blueberries**								
	*C. parvum*					Median	95% CI	
Running water	96.44	91.57	99.86	99.97	99.55	99.55	91.57	99.97
Vinegar	99.95	99.81	99.94	99.33	98.64	99.81	98.63	99.95
Salad spinner	99.99	99.99	99.82	99.39	99.99	99.99	99.38	99.99
	*G. duodenalis*							
Running water	96.07	95.47	97.21	94.01	88.41	95.47	88.41	97.21
Vinegar	94.97	97.23	94.6	92.62	95.04	94.97	92.61	97.23
Salad spinner	95.64	95.04	95.64	98.46	92.75	95.64	92.74	98.45
	*C. cayetanensis*							
Running water	97.22	94.17	97.91	96.63	96.12	96.63	94.17	97.91
Vinegar	99.1	97.02	95.83	99.4	97.37	97.37	95.82	99.40
Salad spinner	99.18	96.11	98.13	99.18	97.73	98.13	96.11	99.18

## Supplementary Materials

The following are available online at https://www.mdpi.com/2304-8158/10/2/481/s1.
