# Supplementary material for: Removal of Parasite Transmission Stages from Berries Using Washing Procedures Suitable for Consumers
Source: Foods. 2021 Feb 23;10(2):481. doi: 10.3390/foods10020481 (PMC7926854; doi:10.3390/foods10020481)
Supplement: Supplementary file 1 [file foods-10-00481-s001.zip › foods-1094607-supplementary.pdf]

Supplementary file 1

1. Standard curve for *Cyclospora cayetanensis* quantification from raspberry spikes

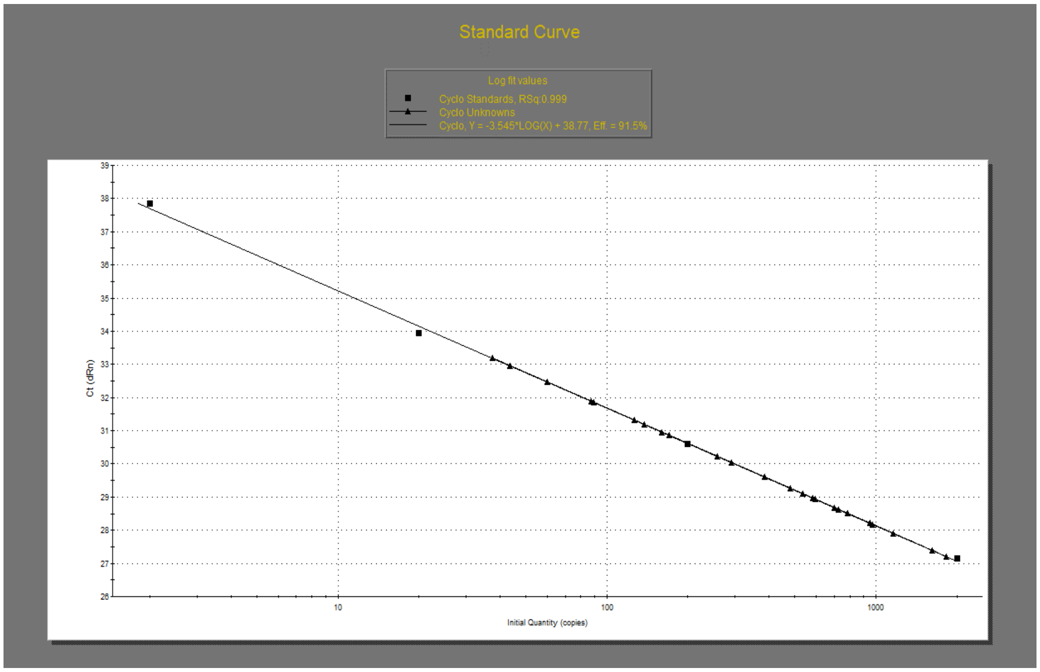

2. Standard curve for *Cyclospora cayetanensis* quantification from blueberries spikes

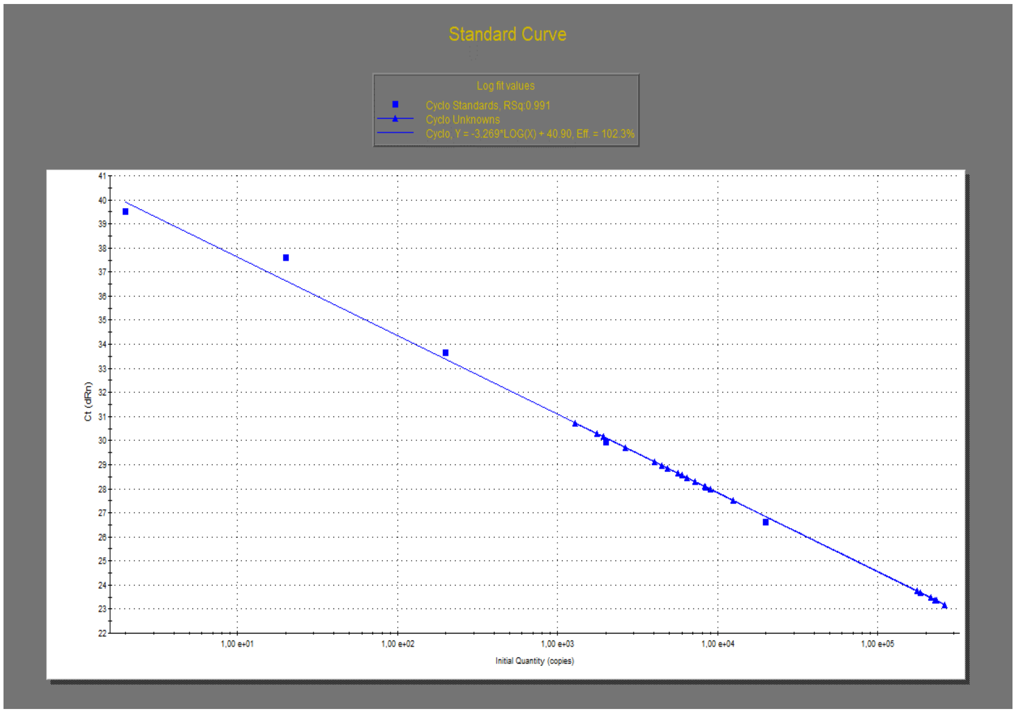

3. Standard curve for *Giardia duodenalis* quantification from raspberries spikes

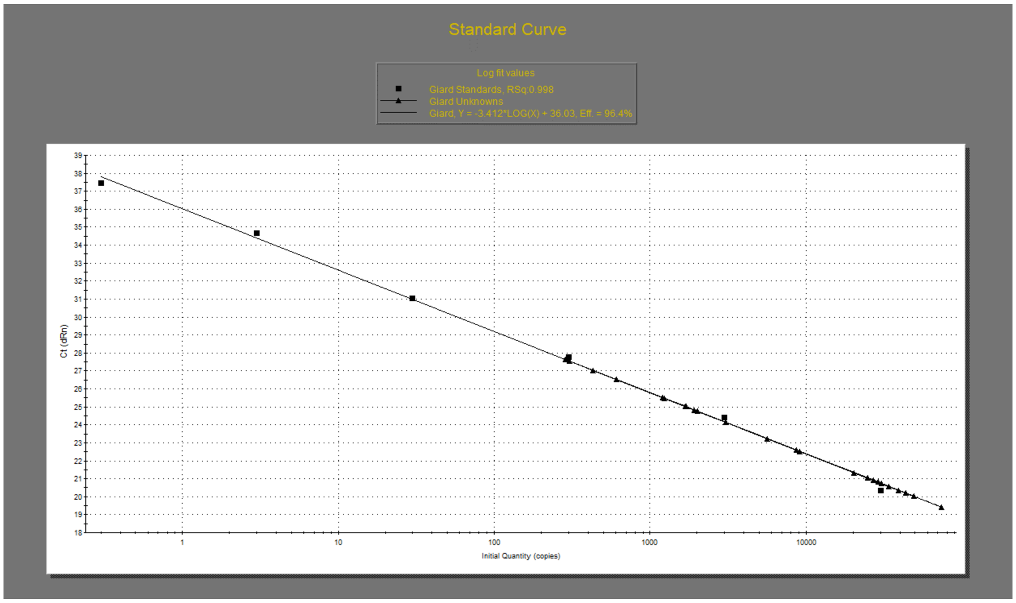

4. Standard curve for *Giardia duodenalis* quantification from blueberries spikes

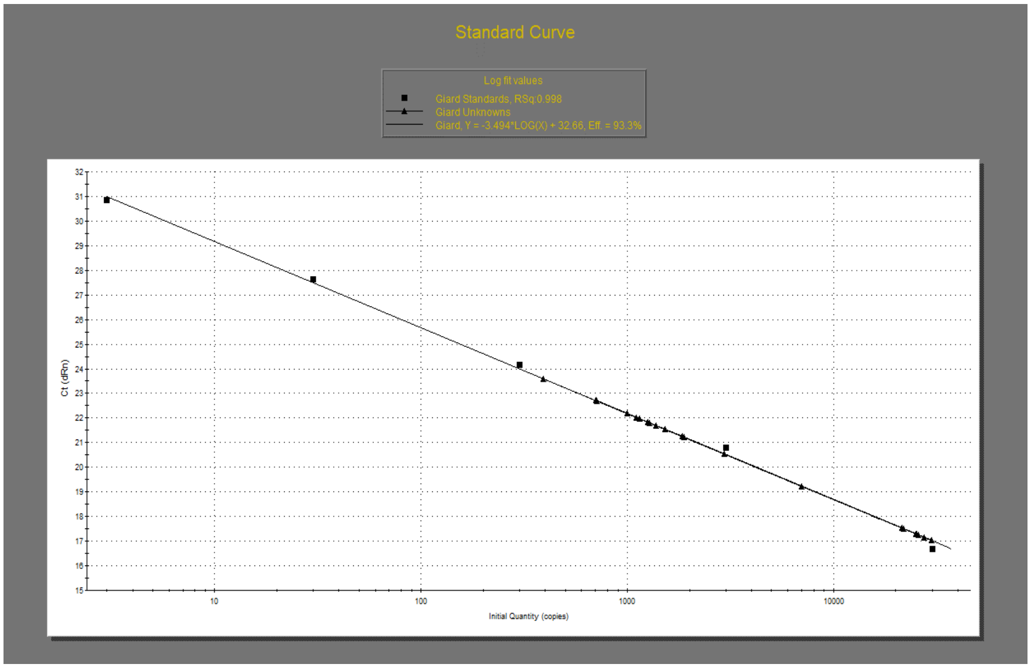

5. Standard curve for *Cryptosporidium parvum* quantification from blueberries spikes

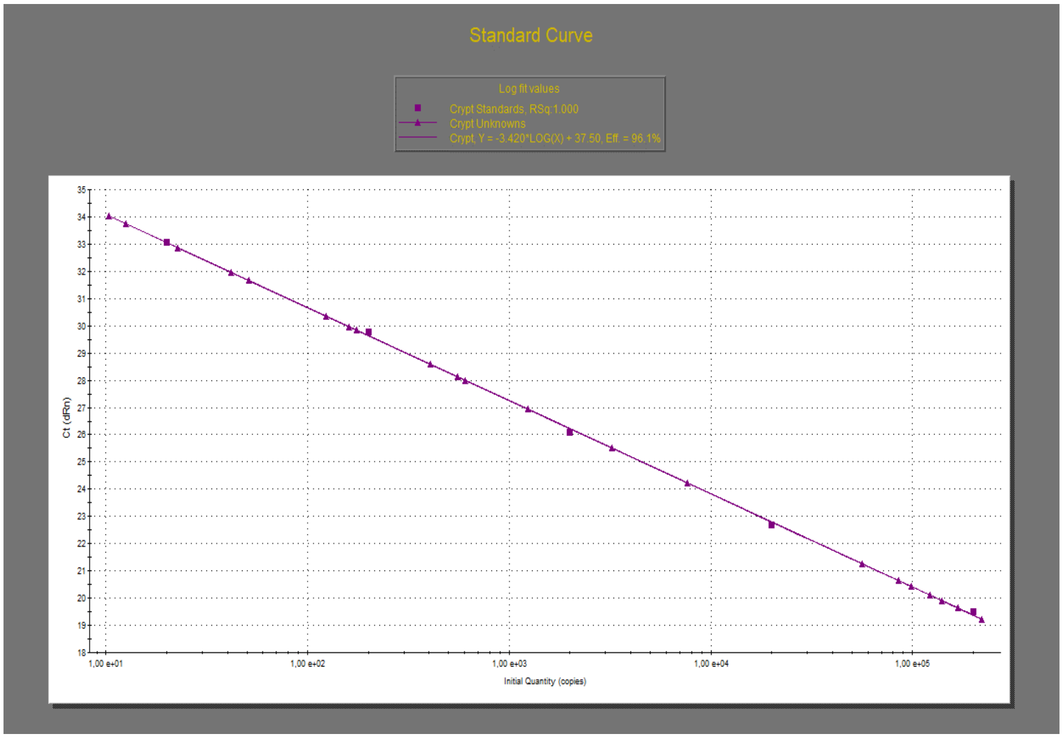

6. Standard curve for *Cryptosporidium parvum* quantification from raspberries spikes

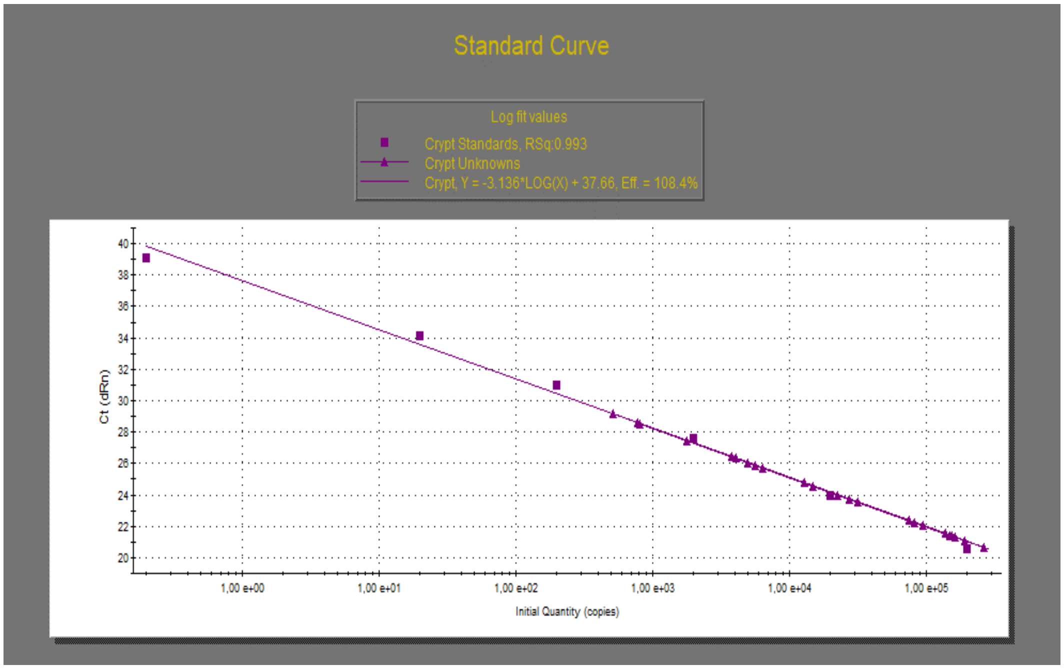

Supplementary file 2.

This file presents the qPCR results and estimated removal efficacy, in percentage, of each washing alternatives. The washing alternatives included Running water (RW), Vinegar (VG), and salad spinner (SP).

Table 1. Detection and quantification of *Cryptosporidium parvum* from blueberries samples.

Well Name	Well Type	Ct (dRn)	Estimated no. of oocysts	% removed
RW-1	Unknown	25.5	3224	96.44
RW-2	Unknown	24.23	7625	91.57
RW-3	Unknown	30.34	124.1	99.86
RW-4	Unknown	32.86	22.77	99.97
RW-5	Unknown	28.59	405	99.55
VG-1	Unknown	31.95	41.9	99.95
VG-2	Unknown	29.83	174.6	99.81
VG-3	Unknown	31.66	51.09	99.94
VG-4	Unknown	27.99	605.8	99.33
VG-5	Unknown	26.93	1234	98.64
SP-1	Unknown	33.75	12.54	99.99
SP-2	Unknown	34.03	10.36	99.99
SP-3	Unknown	29.95	161.2	99.82
SP-4	Unknown	28.11	556.1	99.39
SP-5	Unknown	37.78	0.8316	99.99
C-1	Unknown	21.26	56070	NA
C-2	Unknown	20.42	98510	NA
C-3	Unknown	20.11	121600	NA
C-4	Unknown	20.63	85630	NA
NC	Negative control	No Ct	No Ct	NA

Table 2. Detection and quantification of *Giardia duodenalis* from blueberries samples.

Well Name	Well Type	Ct (dRn)	Estimated no. of cysts	% removed
RW-1	Unknown	22.18	9.99E+02	96.07
RW-2	Unknown	21.97	1.15E+03	95.47
RW-3	Unknown	22.7	7.09E+02	97.21
RW-4	Unknown	21.54	1.52E+03	94.01
RW-5	Unknown	20.54	2.95E+03	88.41
VG-1	Unknown	21.81	1.28E+03	94.97
VG-2	Unknown	22.71	7.04E+02	97.23
VG-3	Unknown	21.7	1.37E+03	94.60
VG-4	Unknown	21.23	1.88E+03	92.62
VG-5	Unknown	21.83	1.26E+03	95.04
SP-1	Unknown	22.03	1.11E+03	95.64
SP-2	Unknown	21.83	1.26E+03	95.04
SP-3	Unknown	22.03	1.11E+03	95.64
SP-4	Unknown	23.6	3.93E+02	98.46
SP-5	Unknown	21.25	1.84E+03	92.75
C-1	Unknown	19.23	6.99E+03	NA
C-2	Unknown	17.53	2.15E+04	NA
C-3	Unknown	17.3	2.50E+04	NA
C-4	Unknown	17.02	3.00E+04	NA
C-5	Unknown	17.28	2.53E+04	NA
NC	Negative control	No Ct	NA	NA

Table 3. Detection and quantification of *Cyclospora cayetanensis* from blueberries samples.

Well Name	Well Type	Ct (dRn)	Estimated no. of oocysts	% removed
RW-1	Unknown	28.55	6.00E+03	97.22
RW-2	Unknown	27.5	1.26E+04	94.17
RW-3	Unknown	28.96	4.51E+03	97.91
RW-4	Unknown	28.28	7.26E+03	96.63
RW-5	Unknown	28.08	8.37E+03	96.12
VG-1	Unknown	30.15	1.95E+03	99.10
VG-2	Unknown	28.45	6.43E+03	97.02
VG-3	Unknown	27.98	9.00E+03	95.83
VG-4	Unknown	30.73	1.29E+03	99.40
VG-5	Unknown	28.63	5.68E+03	97.37
SP-1	Unknown	30.28	1.77E+03	99.18
SP-2	Unknown	28.07	8.40E+03	96.11
SP-3	Unknown	29.12	4.03E+03	98.13
SP-4	Unknown	30.29	1.77E+03	99.18
SP-5	Unknown	28.84	4.89E+03	97.73
C-1	Unknown	29.71	2.66E+03	NA
C-2	Unknown	23.35	2.33E+05	NA
C-3	Unknown	23.68	1.85E+05	NA
C-4	Unknown	23.38	2.29E+05	NA
C-5	Unknown	23.47	2.15E+05	NA
NC	Negative control	No Ct	NA	NA

Table 4. Detection and quantification of *Cryptosporidium parvum* from raspberries samples.

Well Name	Well Type	Ct (dRn)	Estimated no. of oocysts	% removed
RW-1	Unknown	24.79	6.36E+03	94.45
RW-2	Unknown	23.57	1.45E+04	87.37
RW-3	Unknown	23.73	1.29E+04	88.69
RW-4	Unknown	22.45	3.06E+04	73.26
RW-5	Unknown	22.86	2.32E+04	79.69
VG-1	Unknown	23.59	1.42E+04	87.61
VG-2	Unknown	27.76	8.65E+02	99.24
VG-3	Unknown	25.17	4.91E+03	95.71
VG-4	Unknown	26.35	2.22E+03	98.06
VG-5	Unknown	25.31	4.48E+03	96.09
SP-1	Unknown	24.68	6.86E+03	94.01
SP-2	Unknown	24.86	6.07E+03	94.70
SP-3	Unknown	26.96	1.48E+03	98.71
SP-4	Unknown	27.33	1.15E+03	98.99
SP-5	Unknown	22.57	2.82E+04	75.36
C-1	Unknown	20.35	1.26E+05	NA
C-2	Unknown	20.47	1.16E+05	NA
C-3	Unknown	20.3	1.30E+05	NA
C-4	Unknown	20.45	1.18E+05	NA
C-5	Unknown	20.96	8.33E+04	NA
NC	Negative control	No Ct	NA	NA

Table 5. Detection and quantification of *Giardia duodenalis* from raspberries samples.

Well Name	Well Type	Ct (dRn)	Estimated no. of cysts	% removed
RW-1	Unknown	23.23	5.67E+03	87.97
RW-2	Unknown	22.52	9.11E+03	80.67
RW-3	Unknown	25.48	1.24E+03	97.37
RW-4	Unknown	21.03	2.48E+04	47.26
RW-5	Unknown	22.6	8.63E+03	81.67
VG-1	Unknown	26.53	6.07E+02	98.71
VG-2	Unknown	27.65	2.86E+02	99.39
VG-3	Unknown	25.01	1.70E+03	96.39
VG-4	Unknown	27.56	3.04E+02	99.36
VG-5	Unknown	24.76	2.01E+03	95.73
SP-1	Unknown	25.03	1.68E+03	96.43
SP-2	Unknown	24.83	1.92E+03	95.92
SP-3	Unknown	25.52	1.21E+03	97.44
SP-4	Unknown	27.04	4.33E+02	99.08
SP-5	Unknown	24.14	3.05E+03	93.52
CT-1	Unknown	20.35	3.93E+04	NA
CT-2	Unknown	20.81	2.89E+04	NA
CT-3	Unknown	20.02	4.94E+04	NA
CT-4	Unknown	20.2	4.36E+04	NA
CT-5	Unknown	19.41	7.43E+04	NA
NC	Negative control	No Ct	NA	NA

Table 6. Detection and quantification of *Cyclospora cayetanensis* from raspberries samples.

Well Name	Well Type	Ct (dRn)	Estimated no. of oocysts	% removed
RW-1	Unknown	30.22	2.57E+02	68.61
RW-2	Unknown	29.6	3.85E+02	52.97
RW-3	Unknown	28.93	5.95E+02	27.41
RW-4	Unknown	28.63	7.26E+02	11.41
RW-5	Unknown	29.1	5.34E+02	34.88
VG-1	Unknown	31.31	1.27E+02	84.52
VG-2	Unknown	33.19	3.76E+01	95.41
VG-3	Unknown	30.86	1.71E+02	79.18
VG-4	Unknown	31.85	8.95E+01	89.07
VG-5	Unknown	31.19	1.38E+02	83.19
SP-1	Unknown	32.46	6.00E+01	92.67
SP-2	Unknown	31.88	8.78E+01	89.28
SP-3	Unknown	30.96	1.60E+02	80.48
SP-4	Unknown	32.96	4.35E+01	94.69
SP-5	Unknown	30.04	2.91E+02	64.51
CT-1	Unknown	28.68	7.00E+02	NA
CT-2	Unknown	28.51	7.85E+02	NA
CT-3	Unknown	29.26	4.81E+02	NA
CT-4	Unknown	27.91	1.16E+03	NA
CT-5	Unknown	28.18	9.73E+02	NA
NC	Negative control	No Ct	No Ct	NA
